# Rotational Flap to Enhance Buccal Gingival Thickness and Implant Emergence Profile in the Esthetic Zone: Two Cases Reports

**DOI:** 10.2174/1874210601711010284

**Published:** 2017-06-30

**Authors:** Mohammed Jasim AL-Juboori

**Affiliations:** Department of Periodontology, Al-Rafidain University College, Baghdad, Iraq.

**Keywords:** Esthetic zone, Gingival level, Emergence profile, Soft tissue, Flap

## Abstract

**Objective:**

Many techniques have been developed to enhance the gingival thickness, gingival level and emergence profile around the implant in the esthetic zone.

**Introduction:**

In this study, a buccal rotational flap was used to improve the implant site in the esthetic zone and increase gingival tissue thickness.

**Methods:**

Two cases involved the use of a rotational flap during second-stage implant surgery, one case involved the use of a temporary crown with a healing abutment, and another case involved the use of a healing abutment.

**Result:**

The cases were followed up until the final crown was placed. The implant site was improved in 2 cases; the gingival thickness increased, the gingival level was enhanced and the emergence profile was developed.

**Conclusion:**

Many factors affect the results of a rotational flap; some factors are surgical, while others are prosthetic, biological and anatomical.

## INTRODUCTION

The placement of an implant in the esthetic zone requires comprehensive knowledge of anatomy, tissue types, tissue behavior and the healing process, in addition to good skills [1]. From a hygienic point of view, the ideal tissue around the implant is attached keratinized gingiva [2]. From an esthetic point of view, attached keratinized gingiva is not adequate for achieving optimum results in the esthetic zone in patients with a high smile line. A healthy gingival margin level, the existence of interdental papillae, and the emergence profile of the crown are mandatory for achieving an esthetic and harmonious appearance [3]. After tooth extraction, many physiological changes occur in the edentulous site because of keratinized tissue loss, interdental papillae loss, and alveolar gingival thinning [4]. Many techniques have been developed to improve the soft tissue around the implant, such as the AlloDerm roll technique, particulate onlay grafts and connective tissue grafts [5-7].

Augmentation with intraorally harvested connective tissue showed a good result for maintaining the gingival level and thickening of the gingival tissue [8]. Still, connective tissue grafting has some drawbacks resulting from the need for a second surgery site, shrinkage, changes in color, necrosis caused by poor blood supply and the advanced professional skill needed [9-12].

The palatal roll technique is used to enhance the gingival margin thickness when the defect is smaller than 3 mm. This technique can be used in stage two surgery to enhance the labial gingival contour and the implant emergence profile [10, 13]. The palatal roll technique has many advantages and disadvantages Table **1**. This paper reports two cases treated with a palatal rotational flap during the secondary stage. Two cases elaborate the elements underlying the success of the rotational flap technique.

## CASE NO. ONE

**Fig. (1):**
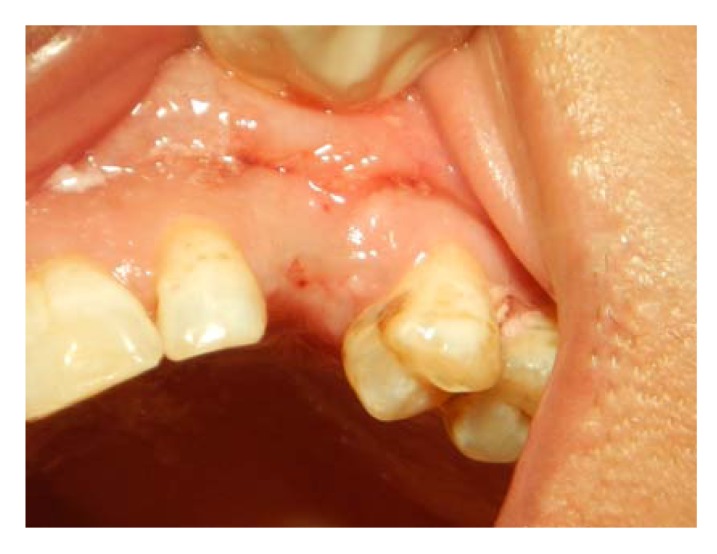
Retained root 23 is extracted, and the socket is left to heal for 2 months before implant placement.

**Fig. (2):**
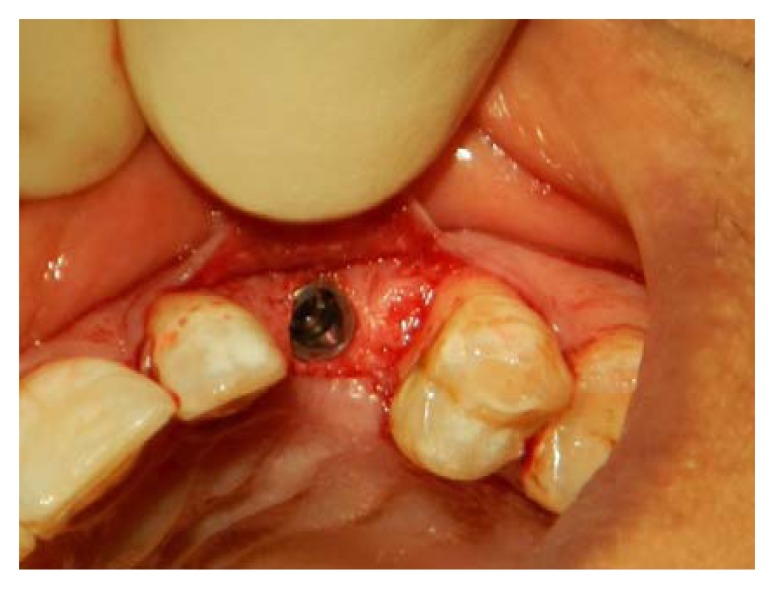
Full-thickness flap is raised using crestal incision with papillary involvement to place an implant in the 23 area.

**Fig. (3):**
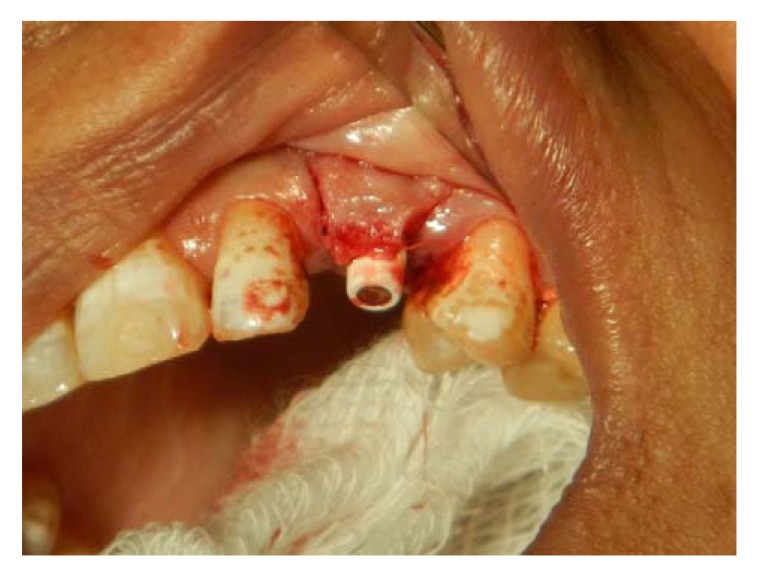
Re-entry surgery is performed 3 months after implant placement and palatal rotational flap performed simultaneously with a temporary abutment and crown. A three-sided flap with a more palatally placed crestal incision and the papillae reserved is created. Two vertical incisions are made to the vestibule so that the flap could be freely rotated.

**Fig. (4):**
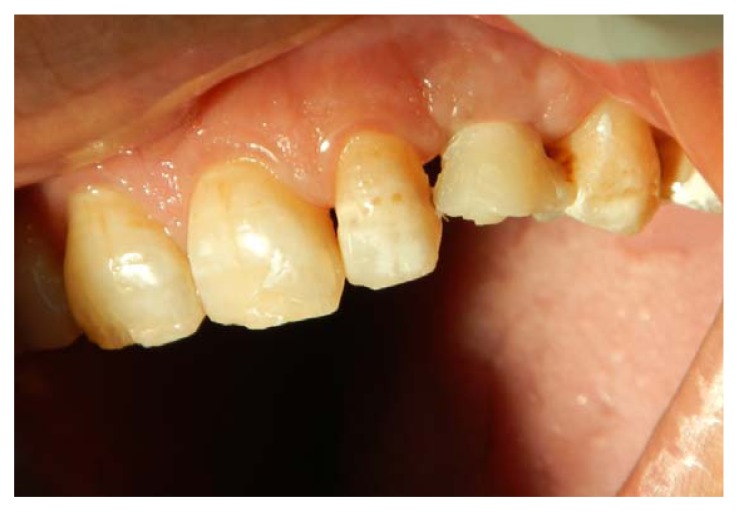
After one month, the crown is modified by adding composite filling material to shape the emergence profile to mimic a natural tooth.

**Fig. (5):**
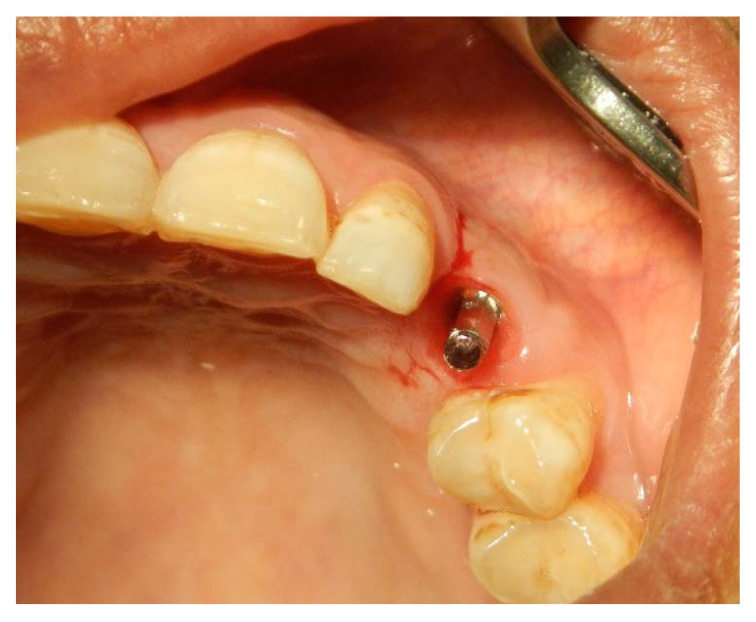
Soft tissue adaptation occurred and an emergence profile was shaped when a provisional crown was used. Bleeding occurred after the provisional crown was removed, which is a sign that the lining mucosa was attaching to the crown structure.

**Fig. (6):**
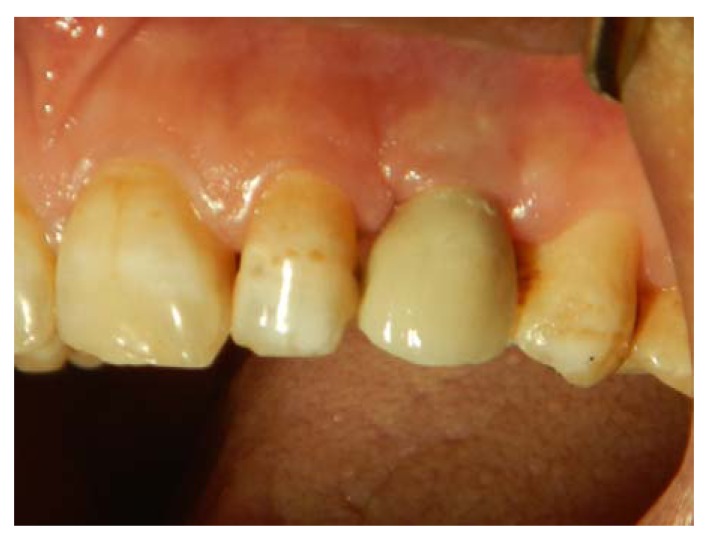
The permanent crown is placed, and gingival bulk is achieved. Still, gingival indentation persists at the vertical gingival incision, and partially loss of mesial and distal papillae is obvious.

**Fig. (7):**
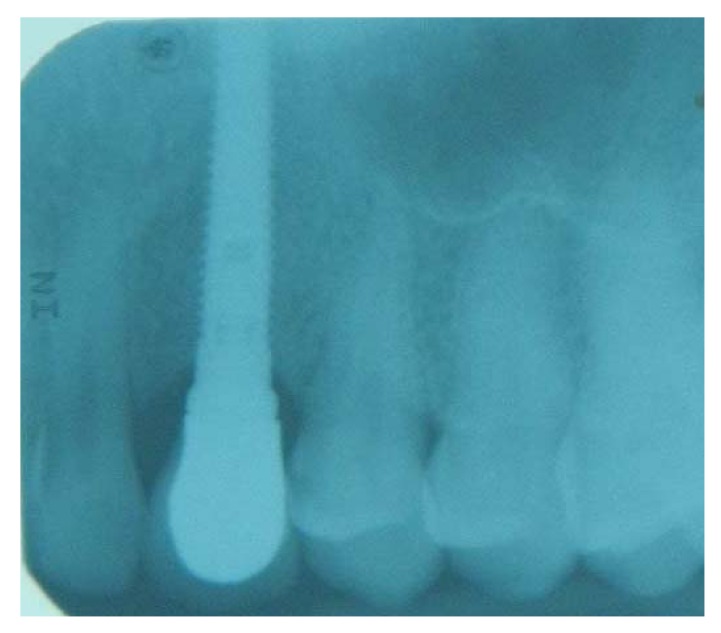
Periapical radiograph at the time the crown was placed shows a good crestal bone level.

**Fig. (8):**
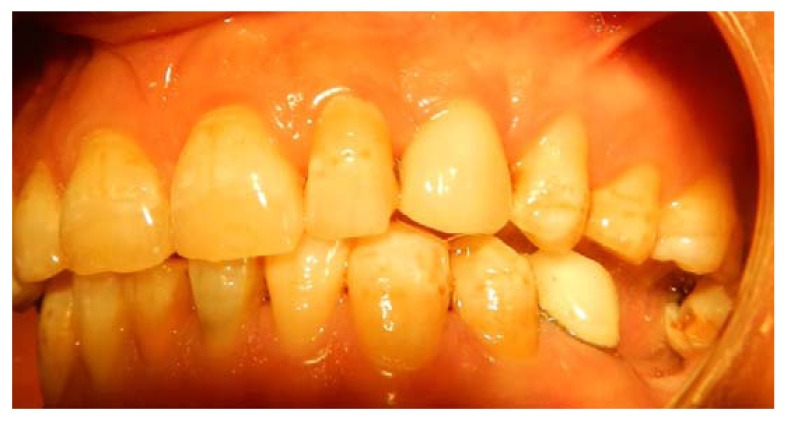
Six months after the permanent crown is placed, the papillae regenerate and fill the gap, and the indentation scar from the vertical incision starts to disappear.

A 35-years old patient attended our clinic with a non-restorable retained root of 23. The treatment plan discussed with patient involved removing the retained root and placing an implant after 2 months to allow for soft and bony tissue healing. Two months after (Fig. **1**) the implant was placed, a full-thickness envelope was raised with the adjacent papillae. An osteotomy was performed according to manufacturer recommendations, and an implant 16 mm in length and 3.7 mm in diameter was placed (Fig. **2**). The implant was placed with moderate stability (torque 25 Ncm). The cover screw was placed, the flap was repositioned, and the implant was completely covered. The implant was left to heal for 2 months, after which re-entry surgery was performed by raising a three-sided flap that excluded the adjacent papillae. A palatal crestal incision was made, the cover screw was removed, and a temporary abutment with a provisional crown made of composite was prepared. The provisional crown was small in shape (diameter and length) with open contact with adjacent teeth to allow soft tissue growth and thickening. The temporary abutment was torqued into place, and the crown was polished and cemented onto it. After de-epithelization with scalpel the flap was rotated inward with the keratinized mucosa facing the temporary crown and simple interrupted suturing with vicryl suture was performed to fix the rotated flap against the crown and the palatal tissue (Fig. **3**). The temporary crown was adjusted to be kept out of occlusion during centric movement and excursion. After one week, the suture was removed, and the wound healed uneventfully. After one month, the crown removed and reshaped by increasing the diameter; it was still kept out of occlusion (Fig. **4**). After 3 months with complete soft tissue maturation, the temporary crown was removed to reveal an excellent emergence profile (Fig. **5**), the temporary abutment was removed, and impression coping was placed to make an impression at the bone level. The permanent crown was placed, excess cement was removed, and occlusion was adjusted (Fig. **6**). After one month, the patient was followed up and radiographed (Fig. **7**), and the papillae were still partially filling the interdental space. The patient was seen again after 3 months, at which time the papillae were almost filling the interdental space and the scar was faded (Fig. **8**).

## CASE NO. TWO

**Fig. (9):**
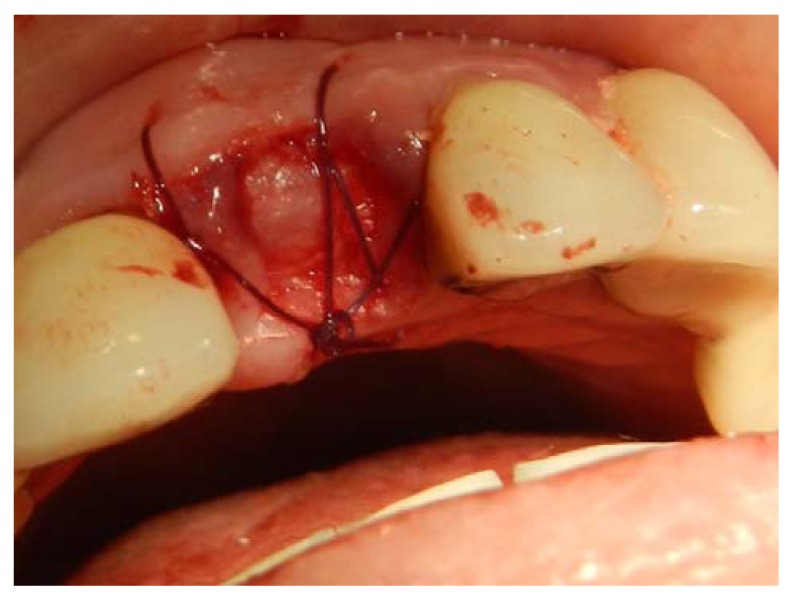
Socket grafting after 21 extraction. The bone graft is covered with a collagen membrane and secured with sutures.

**Fig. (10):**
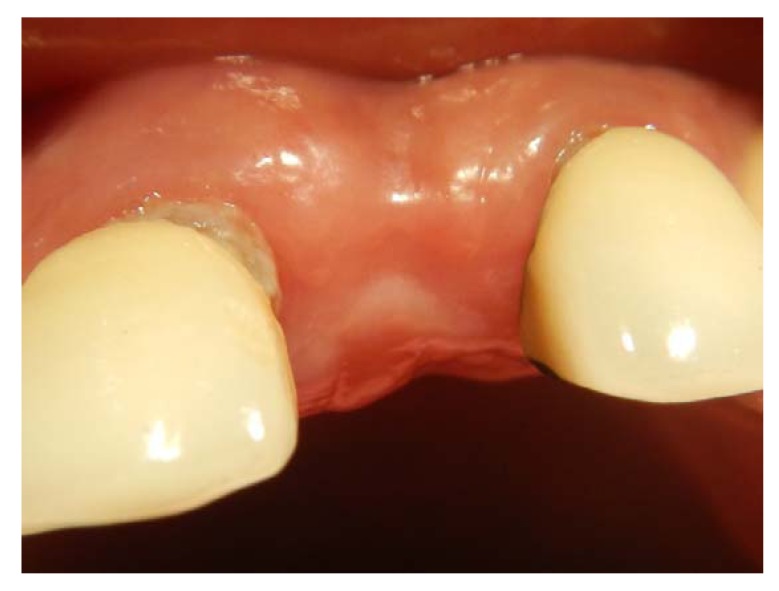
Buccal contour concavity after 3 months period of implant placement.

**Fig. (11):**
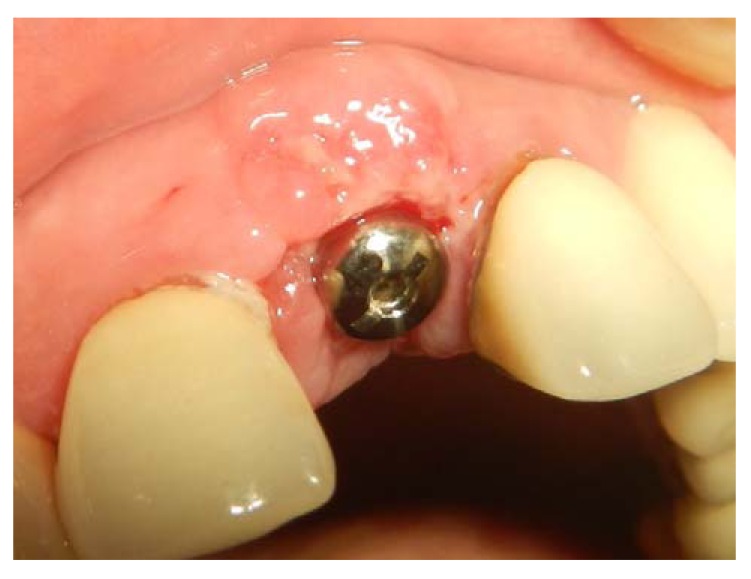
Healing one week after the palatal rotational flap is performed. Overcorrection is mandatory to compensate for soft tissue remodeling.

**Fig. (12):**
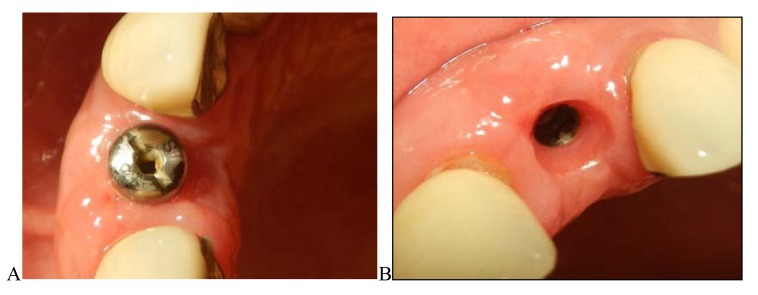
Soft tissue healing one month after rotational flap. **A**, the normal contour of the implant buccal gingiva; **B**, thick and healthy gingival tissue around the implant after the healing abutment is removed.

**Fig. (13):**
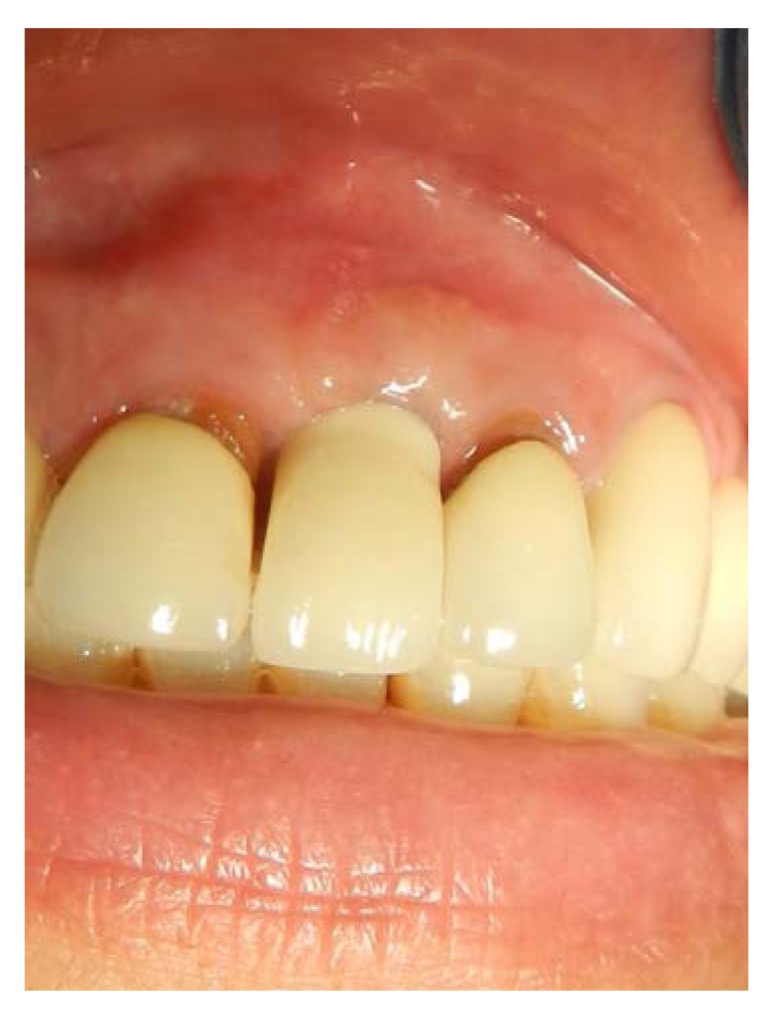
A permanent crown with a customized abutment is fabricated, a marked gingival augmentation can be appreciated.

**Fig. (14):**
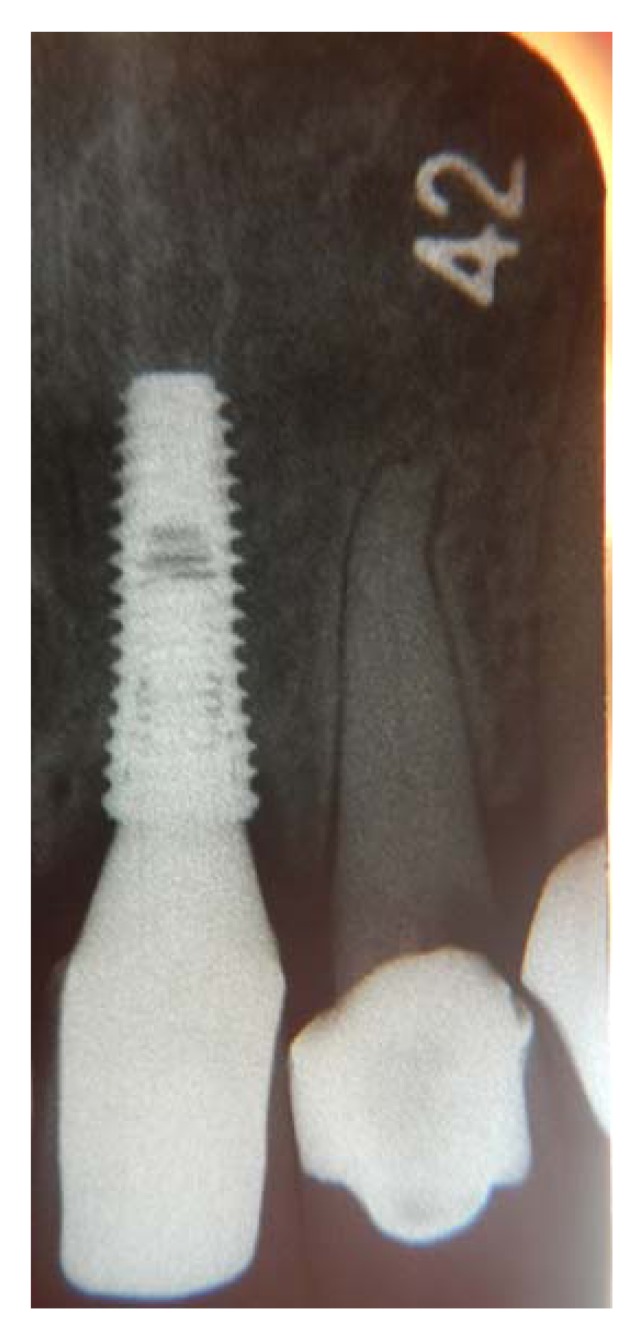
Periapical radiograph at the time the crown was placed shows a good crestal bone level.

A 56-years old woman attended our clinic complaining of a mobile tooth 21. Upon examination, the tooth presented grade 2 mobility, gingival recession and overeruption. The adjacent teeth suffered from gingival recession, and the patient had an average smile line. The patient was advised to have the tooth removed and an implant placed. The tooth was removed, and when the socket was checked, buccal bone loss was found. A cancellous bone graft was placed, covered with a collagen membrane (double layer) and secured with mattress sutures (Fig. **9**). The extracted tooth was replaced temporarily with a Maryland bridge. After one month, the socket was completely covered by keratinized mucosa. After 4 months, the grafted site was exposed, with thick, keratinized gingival tissue formation, and an adequate amount of bone had formed to place a 3.6 x 10-mm implant. The implant was submerged and left to heal for 3 months. A horizontal depression was observed in the center of implant site (Fig. **10**). After that, the decision was made to expose the implant using a palatal crestal incision with 2 incisions extending from the crestal incision towards the labial gingival tissue and papillae preservation. No vertical cut was made on the labial keratinized tissue to avoid scar formation. The implant was exposed, the cover screw was removed, and a healing abutment was placed. After de-epithelization with scalpel the flap was rotated inward, tagged underneath the labial gingival tissue and secured to the palatal mucosa with sutures. After one week, the sutures were removed. The wound healed uneventfully, and ample gingival tissue thickness and even overbulging was observed (Fig. **11**). The wound was left to heal for 4 weeks (Fig. **12**), and then an impression was taken with impression coping. A customized abutment with porcelain fused to metal crown was fabricated (Fig. **13**) and cemented on, and the occlusion was adjusted for centric and eccentric movement. Patient was followed up by radiograph that show stable crestal bone level (Fig. **14**).

## DISCUSSION

Proper soft tissue management is a key to success in implant dentistry, especially in the esthetic zone. Soft tissue thinning and horizontal bone resorption are common changes that occur after tooth extraction [14-18].

These changes will affect the gingival level, crown emergence profile and gingival contour [4]. In the first case, a delayed implant placement regimen was followed, while in the second case, socket preservation was performed. In the second case, socket preservation did not prevent the occurrence of horizontal bone reservation that required a rotational flap during the second stage to address the buccal defect problem.

In both case reports, a buccal gingival rotational flap has many advantages (Table **1**) when was used to increase the thickness of the gingiva and improve the crown emergence profile. Using a local flap will avoid second-site surgery when the connective tissue graft is harvested from the palate [9, 10].

Moreover, there is no shrinkage of the mucosa or changes in color when the gingival mucosal graft is placed at the gingival defect site [8, 9].

Another advantage is that there is no risk of graft loss or necrosis resulting from a loss of blood supply to the graft as the rotation flap provides and maintains the blood supply to the rotated part of the gingiva. Color matching is another advantage, as the gingival tissue surrounding the implant will stay in place. This procedure offers more advantages, requires less skill and provides a shorter healing time compared with a connective tissue graft when only 4 weeks on the second case was enough to obtain tissue healing and maturation. This technique can provide the bulkiness that is needed to achieve better esthetics while hiding the mild bone resorption and improving the implant’s esthetic contour. Additionally, it increases the thickness of the gingiva to provide more soft tissue stability around the implant for the long term when compared with AlloDerm [8].

However, when using the rotational flap, some considerations must be followed to achieve better results. To achieve predictable results, certain elements should be followed and achieved (Table **2**). First, enough flap tissue thickness needs to be rotated to improve the gingival contour. Second, keratinized tissue should be present at the buccal site before the rotational flap is started (presented cases have more than 5mm buccal) as the rotational flap will not change the biotype of the gingiva if non-keratinized mobile tissue is found at the labial side. Third, a papillae-preserving flap is recommended in the esthetic zone to prevent the interdental papillary loss that occurred in the first case, when the crestal incision included the interdental papillae with the flap [19]. An interdental papillae-preserving flap should be used in the first (implant placement) and second (re-entry) surgeries [20].

Interdental papillae are not surgically recreated, but they are surgically maintained. In other words, the surgical procedure cannot regenerate the papillae, but the surgery should be conservative and should not include the papillae from the first surgery [20]. This technique does not improve the interdental papillae level as the papillae depend on the crestal bone-contact point distance [21].

Fourth, it is important to avoid making a vertical incision in the keratinized mucosa that can cause the formation of an unpleasant scar, which was obvious in the first case; instead, the surgeon can use a pouch and tuck the rotated flap inside [10]. Using a provisional crown will improve the gingival emergence profile to mimic a natural tooth profile [19].

The thickness of the rolled gingiva is crucial in this procedure to create more bulge and thickness on the buccal side of the implant [10].

## CONCLUSION

The use of the rotational flap technique can improve and address horizontal concavity of the buccal bone and enhance the gingival margin level around the crown.

## Figures and Tables

**Table 1:** Palatal flap roll technique advantages and disadvantages.

**Disadvantages**	**Advantages**
1-Does not change the gingival biotype, 2-Tissue loss may occur at the implant site, 3-Not applicable for multiple implants, 4-Does not improve the interdental papillae level, 5-May leave a scar at the esthetic zone	1-Achieves a better emergence profile, 2-Less scar tissue formation, 3-Stable gingival margin, 4-No secondary site of surgery, 5-Mantains the blood supply to the rotated part, 6-Shorter healing period and faster tissue maturation, 7-No need for advanced professional skills, 8-Cost effective, 9-Tissue harmony around the implant

**Table 2:** Factors, procedure and elements that contribute in successful rotational flap technique.

**Surgical**	**Prosthetic**	**Biological**	**Anatomical**
1-Papillae-preserving flap	1-Proper implant position (not ending with a ridge lab crown)	1-Oral mucosal tissue changes according to the type of surface facing the implant	1-Adequate width of keratinized mucosa on the buccal aspect
2-Avoiding a vertical cut on the keratinized mucosa	2-Concave provisional crown at the cervical area		2-Adequate thickness of the rotated flap (>1.5 mm)
3-More palatal placement of the crestal incision	3-Smooth crown surface		
